# Explaining consumer motives to purchase in the informal economy

**DOI:** 10.1371/journal.pone.0258686

**Published:** 2021-10-15

**Authors:** Ioana Alexandra Horodnic, Colin Charles Williams, Jan Windebank, Adriana Zaiț, Claudia Ioana Ciobanu

**Affiliations:** 1 Faculty of Economics and Business Administration, Alexandru Ioan Cuza University of Iași, Iași, Romania; 2 Management School, University of Sheffield, Sheffield, United Kingdom; 3 School of Languages and Cultures, University of Sheffield, Sheffield, United Kingdom; 4 Faculty of Civil Engineering and Building Services, Gheorghe Asachi Technical University of Iași, Iași, Romania; Szechenyi Istvan University: Szechenyi Istvan Egyetem, HUNGARY

## Abstract

Usually, studies on the informal economy focus mainly upon those working in the informal economy (supply side). However, many exchanges in the informal economy are initiated by purchasers asking how much a good or service costs if paid cash in hand. Therefore, the aim of this paper is to advance understanding of who make purchases in the informal economy and the reasons of the consumers making these purchases (demand side). Two potential explanations are evaluated. Firstly, consumers are explained as rational economic actors seeking a more convenient deal or profit maximisation (i.e., lower price or better value for money), making purchases from the informal economy due to the lack of availability of the product or service they need on the formal market, or they make such purchases involuntarily, due to the lack of perfect information necessary to make a fully rational economic decision when purchasing. Secondly, the consumers are portrayed as social actors pursuing community help. Using a multilevel mixed-effects logistic regression analysis on a 2019 Eurobarometer interviews in 27 EU member states and the UK reveal how the prevalence of these motives significantly varies across populations and regions. The theoretical and policy implications of the findings are discussed in the concluding section.

## 1. Introduction

Until now, most studies on the informal economy have investigated the supply-side of those who work in the informal economy, examining what types of work they undertake [1, 2], the socio-demographic profile of informal workers [3–6], their motives for working in the informal economy [7, 8] or the drivers of informal economy, including institutional drivers [9], economic drivers [10] and socio-environmental drivers [11, 12]. The demand-side perspective has received scant attention. Indeed, the few studies conducted to date report data from 2007 and 2013 [13–17] or, if more recent data, they only analysed a single economic sector [18, 19]. Therefore, little current knowledge exists on consumers of goods and services from the informal economy. As such, the aim of this paper is to provide a contemporary evaluation of who makes purchases in the informal economy and the reasons behind these purchases. The importance of understanding this subject is that more than 60% of the global workforce have employment in the informal economy [20, 21], displaying that this is not some minor segment of consumption but a major sphere of production and consumption across the world. Therefore, to omit to study consumer behaviour in this large and extensive realm is to ignore a major source of the goods and services consumed in the world and an important facet of contemporary consumer culture. As Venkatesh and Peñaloza [22] assert, despite the prevalence of the informal economy and its important social and economic role across the globe, there is little or no research on informal markets from a marketing perspective apart from a study by Arnould in 1995 [23].

This lacuna of studies on the informal sector from a marketing perspective is also documented by Arellano [24] who argues that research on the informal sector is conducted by economists and sociologists who seek to explain this market from a legal, social or economic perspective, and that it fails to focus upon the behaviour of those participating in the market exchange, such as the behaviour of the informal retailers. More recently, Viswanathan et al. [25] warns that the marketing theory of exchange is developed and derived from researching and studying formal markets governed by formal institutions and the applicability of these findings to other types of market, such as informal markets, needs to be treated with caution. Thus, despite the prevalence of the informal exchange of goods and services in both developing and developed countries, little is known so far about consumer behaviour in this large market. This paper, therefore, aims at filling this gap.

To fill this significant gap in the literature and to advance understanding of informal markets, the next section outlines some potential explanations of why consumers make purchases in the informal economy. Consumers are portrayed firstly, as rational economic actors either seeking profit maximization, purchasing from the informal market due to the lack of availability of the goods and/or services on the formal market, or taking the decision to purchase from the informal market because they were not aware that the product or service they buy belongs to the informal economy and therefore, they lack the full or perfect information necessary to make a fully rational economic decision when purchasing. Secondly, purchasers are portrayed as social actors engaging in such behaviour to help a friend or a family member (social and/or redistributive motives). As such, theoretically, this paper’s contribution is to extend the rational economic theoretical perspective towards purchases in the informal economy by investigating motivations beyond the profit maximisation, namely the rational decisions driven by the lack of availability on the formal market or the lack of the complete information about the purchase. To evaluate the relevance of these explanations in the contemporary period, the third section then introduces a 2019 Eurobarometer survey involving 27,565 face-to-face interviews which evaluates purchasers of informal goods and services and their rationales in the 27 member states of the European Union and the UK. The fourth section outlines the results, followed by the fifth and sixth final sections that set out both the advances made in theoretical understandings of consumer behaviour in the informal economy as well as the policy implications of these findings.

At the outset, the definition of the informal economy employed in this paper must be clarified. Reflecting the most dominant view amongst both practitioners and academics, the informal economy refers to paid exchanges which are legal in all respects except the fact that they are hidden or unreported to the state to circumvent the tax, social security and/or labour laws [26–28]. If the good and/or service is not legal in any other respect, it is not part of informal economy but rather it belongs to the wider sphere of the criminal economy. This includes illegal goods (e.g., illegal drugs) or services (e.g., prostitution in some countries). Even if it might be argued that consumers will not know if the supplier declares the income to the state or not, and thus whether they made a purchase in the informal economy, this does not represent an issue here because this paper looks equally to transactions where the consumer knowingly perceives themselves to be participating in, or deliberately initiating exchanges in the informal economy, as well as transactions where the consumer realised only afterwards.

## 2. Explaining purchases in the informal market system

For much of the twentieth century, the informal economy was commonly explained as a leftover from a previous mode of production and consumption and widely viewed as steadily disappearing [29, 30]. Given this, there were few reasons to focus upon this sphere. Since the turn of millennium, however, more attention has been given to the informal economy. This has resulted firstly from the recognition that most workers globally have their main employment in the informal economy [20, 31] and secondly, the recognition that this sphere has substantial negative impacts on governments, economies, workers and consumers alike. Governments lose tax revenue and regulatory control, formal businesses suffer unfair competition, and workers in the informal economy lack social protection and other rights deriving from an employment contract [32]. The consumers in the informal market system, meanwhile, are exposed to risks. Firstly, if the product or service does not satisfy their expectations and the promised quality, the consumer has no legal recourse. In addition, these purchases are not covered by any insurance, so the consumer has no guarantees in relation to their purchase, and has no control or knowledge on whether the health and safety regulations are met [1]. Consequently, it has been widely recognised as important to understand the reasons for making purchases in the informal economy and how this behaviour can be discouraged.

What, therefore, determines that individuals make purchases in the informal economy? Until now, the focus has been largely upon explaining the supply-side, such as who works in the informal economy and why they do so [8, 33–36]. Few have studied the informal economy from the demand-side (for notable exceptions, see [13–19]). Here, in consequence, we seek to understand who makes purchases in the informal economy and why they engage in this behaviour. To do this, and based on these earlier studies, we evaluate various possible theories for explaining why consumers are making purchases in the informal economy, each suggesting very different explanations and policy approaches.

### 2.1. Rational economic actor theoretical perspective

#### 2.1.1. Looking for a more convenient deal

The dominant theoretical perspective explaining participation in the informal economy is that the individuals are rational economic actors aiming for financial gains. This approach has its roots in the classic work of Bentham [37] whose utilitarian theory portrays individuals as rational actors which decide whether to engage in an illegal activity by weighing up the benefits and the risk associated with that activity. As such, individuals disobey the law if the expected sanction and risk of detection is lower than the financial benefits. This utility maximizing view was applied to the informal economy in the early 1970s by Allingham and Sandmo [38] and it has become the dominant view on explaining the participation in the informal sector. On the one hand, those working informally representing the supply side of the informal economy are commonly depicted as rational economic actors who engage in informal work motivated by financial benefits [32, 39], whilst on the other hand, consumers or those making purchases in the informal economy are considered to do so purely to enjoy a lower cost which compensates them for any potential drawbacks [40, 41]. Similarly, from a rational economic view, consumers will be more likely to engage in informal transactions if they can obtain better value for their money. As such, they will do so if they can obtain a faster delivery or provision or if the quality of the goods and services provided is better [14]. Therefore, consumers will continue making purchases in the informal economy as long as it is more convenient either due to a lower price or due to obtaining better value for money (i.e., faster provision and/or better quality of provision).

However, in more recent years, other theoretical perspectives have emerged that question the validity of the profit maximisation perspective that those making purchases in the informal economy are always and exclusively motivated by lower prices and financial gain.

#### 2.1.2. Having no other choice

Another explanation for consumers’ purchasing from the informal economy is that these transactions exist due to imperfections in the formal economy. For example, suppliers of informal work, are often argued to willingly exit the formal economy because of the problems that they confront when trying to work formally, such as complicated registration processes, high tax levels and burdensome regulations even or especially for small and occasional paid activities [42–44]. It can be similarly argued that those consumers making purchases in the informal economy make a rational decision when they turn to this sphere due to the poor provision or lack of availability on the formal market, giving the consumer no choice other than to engage in transactions in the informal economy. This can be exemplified by the case of nannies in Romania where the state does not cover any childcare until the child is three years old, making the parents who return to work and cannot afford private childcare to informally employ members of family, neighbours or friends [45].

#### 2.1.3. Involuntary purchases

Finally, it can be asserted that not all transactions in the informal realm are made knowingly by consumers as the above perspectives assume. It might be the case that at least some transactions in the informal economy are involuntarily, the consumer not being aware that the purchase they made belongs to the informal market and therefore lacking the perfect information needed for a fully rational decision. Akin to the supply side, when the workers violate the labour laws or the regulations related to tax and social contributions unintentionally, not being aware of all the up to date regulations in place [46, 47], consumers might involuntarily participate in informal transactions and not be aware until after the purchase has been made and no receipt or invoice was issued, even if requested by the customer.

### 2.2. The social actor theoretical perspective: Mutual aid ends

Recent years have seen the emergence of a social actor theoretical perspective which transcends the view that the individuals are rational economic actors balancing the cost and the benefits of engaging in a specific activity. The social theoretical perspective is rooted in the view adopted by those criticising the narrow view of monetary exchange as being always profit-driven employing a post-capitalist perspective. As an alternative, a broader depiction of monetary exchange has been adopted which recognises the multiple rationales behind exchanges, including social motivations which are often more important than profit-making rationales [48–50].

Adopting this view, a small body of work on the informal economy has started to display how exchanges in the informal economy are frequently by and for close social networks (family members, friends, neighbours, working colleagues and acquaintances) driven by social ends rather than for profit-motivated rationales [51, 52]. As such, those making purchases in the informal economy are viewed from this perspective as social actors and not rational economic actors seeking financial gains. For instance, close social relations are seen to be paid for goods or for doing an activity (e.g., some gardening work, babysitting) so that they can be given much needed money (e.g., when the person supplying the activity is unemployed), without involving the idea of charity, which would very likely stop the person in need from accepting the help offered [53]. These purchases, therefore, are more like mutual aid or community aid than transactions driven by profit [28, 52, 54]. This social actor theoretical perspective, therefore, provides a direct challenge to the rational economic actor theoretical perspective which views participants as mainly seeking financial gain or making the decision to purchase from the informal economy based on the constrains of the formal market (i.e., lack of availability) or because they lack the perfect information about their purchases to be able to make a fully rational decision.

In sum, for fully understanding consumer behaviour, it is important to evaluate both the knowingly and intentional purchases (i.e., due convenience in terms of lower price or better value for money, due to the desire to help a close social contact or because the product or service is not available on the regular formal market) as well as the involuntary purchases.

To evaluate the validity of these competing potential explanations for consumers making purchases in the informal economy in the contemporary period, attention now turns to methodology and results.

## 3. Methodology

To investigate who consumes in the informal economy and why, we used the results of special Eurobarometer survey no. 498 [55], which involved 27,565 face-to-face interviews conducted between 11^th^ and 29^th^ September 2019 across the 27 member states of the European Union (EU-27) and the UK (the data is publicly freely available; for details: [55]). The interviews were conducted face-to-face with adults aged 15 years and older in the respondents’ home and in their mother tongue on the behalf of the European Commission, fulfilling the ethical and data protection requirements. The sample size varied from 500 individuals in small countries to more than 1500 in larger countries. The multi-stage random probability sample design was employed, as well as a weighting scheme, ensuring the match between the responding sample and the population on gender, age, region and degree of urbanisation. As such, for the univariate analysis we weighted the results following the recommendation from the Eurobarometer methodology as well as the dominant view from the wider literature [56]. However, for the multivariate analysis, reflecting the debate over whether a weighting scheme should be used [56–58], the weighting scheme was not employed.

The face-to-face interviews used a gradually approach from to the more sensitive questions. As such, it started with questions about the broad view of the respondents towards informal economy and then, move to questions on whether the respondents made purchases of goods and services in the informal economy in the past 12 month, and if so, what was the rationale behind these purchases. Finally, the questions on informal economy closed with direct questions on participation in informal economy as suppliers of good and services. This paper focuses upon the demand-side questions analysing who make such purchases from the informal economy and the reason of doing so.

Utilizing the hierarchical nature of the data (individuals nested within countries), a multilevel mixed effects logistic regression analysis has been used for analysing who is the consumer in the informal market and why these transactions occur. As such we used five different dependent variables. The first dependent variable is a dichotomous variable displaying who made purchases from the informal market. The rest of the dependent variables, apply only to those who answer positively to the question of whether they purchased any good or service from the informal economy and represent each reason that might have determined the respondents to engage in the informal economy, namely: acquiring from the informal economy because it is more convenient, acquiring from the informal economy due to their absence on the formal market, making this type of purchase involuntarily (realising only afterwards that it was informal market) or acquiring from the informal economy due to social and/ or distributive reasons.

The independent variables used as control were extracted from previous research in informal economy [14, 15, 17–19, 52, 59–62] and include: the individuals`tax morale, gender, marital status, occupation, difficulties in paying bills, having a child or not, area of residence and EU region. The categories of these variables are displayed in the regression tables and the definitions and descriptive statistics in S1 Appendix.

The analysis has been conducted in two steps. The first stage was to decide whether the data used requires a multilevel analysis by estimating the baseline random intercept model with no explanatory variable. The likelihood-ratio test for the null hypotheses according to which there are no variances in terms of who make purchases in the informal economy and/ or the motives of doing so explained at country level can be rejected. As Table 1 displays, the analysis indicates that over 7% of the variance in the propensity of individuals to have made a purchase from the informal economy was accounted for at country level (Wald = 12.8147, df = 1, p < 0.01), indicating significant variation between countries in the prevalence of purchases from the informal sector. The lowest variation (2.88%) between countries is observed when the consumers made these purchases due to poor formal provision. The results in Table 1, therefore indicate that the multilevel mixed-effect regression is the more suitable method for analysing the data.

**Table 1:** Baseline random intercept model–no explanatory variables.

	Wald Test	Variance partition coefficient (VPC)
M1—Purchasing undeclared goods and services	12.8147***	0.0768
M2a–Reasons: More convenient	9.2860***	0.0474
M2b–Reasons: Lack of availability on regular market	4.6019**	0.0288
M2c–Involuntary purchase (realised afterwards it was informal market)	10.3650***	0.1068
M3—Reasons: Social and/or redistributive reasons	9.0909***	0.0543

*Notes*: df = 1

*** p<0.01

** p<0.05.

*Source*: author`s calculations based on data from Special Eurobarometer 498 –Wave EB92.1, Undeclared Work in the European Union, Fieldwork—September 2019 / Publication date—February 2020 (European Commission, 2021).

The second stage involved including individual level variables into the models in order to examine their association with consumers’ motives. We have taken three steps to ensure the robustness of the models including: cross-tabulation of the independent variables against the dependent variables, checking for the multicollinearity issues and model calibration. Furthermore, in order to ease the understanding of the most important results of the regression analysis and to graphically display the differences in the consumer motives in respect with EU regions and the level of individual tax morality, a ‘representative’ European consumer is portrayed based on the mean or modal values of the control variables across the EU-27 and the UK. As such, the ‘representative’ European consumer in the informal economy is a manual worker man, aged 45 years old who is cohabitating with a partner, lives in a two-person household with no children, in a small or middle sized urban area located in Western Europe and who never or almost never face any difficulties in paying the household bills and has a tax morality of 7.9 (out of 10).

Considering the potentially sensitive topic involved, before commencing to the results the reliability of the data used needs to be discussed. Based on the interviewer`s rating, the finding is that the participant cooperation was fair or excellent in about 91% of the cases and average in 8% of the cases. In only 1% of cases the cooperation was declared as bad but rather in relation to engagement in undeclared work (supply-side) and not on purchasing from the informal economy. Having clarified this, the paper continues with analysis of the results.

## 4. Results: Explaining consumer purchases in the informal economy

Analysing the prevalence of transactions on the informal market, the finding is that 10% of the participants from the EU-27 and the UK stated that during the past 12 months they made purchases of goods and services in the informal economy. Some 1 in 10 participants, therefore, self-reported that they had made purchases in the informal economy in the past year. The percentage is slightly higher than in 2007, when 9% of the survey participants reported purchases from the informal market [14, 15]. However, given the sensitivity of the topic, this represents a lower-bound estimate considering that even under anonymity, not all individuals are open or willing to talk about illegal practices [8, 63]. Important to mention is that 16% of those making purchases in the informal economy did so involuntarily, realising that the purchase was from the informal market only afterwards. As Table 2 displays, the predisposition of consumers to acquire goods and services in the informal economy, however, is also unevenly distributed across EU regions. The highest percentage of people purchasing undeclared goods and services is displayed in Nordic Nations, 14%, while the lowest is displayed in Western Europe, 9%. However, and perhaps not surprisingly, these two regions are also the regions where it was reported the highest share of consumers who realised only afterwards that the purchase belong to informal economy (20% of the consumers in Nordic nations and 19% of the consumers in Western Europe). The findings are in line with previous research on the informal economy, with higher percentages of people engaging in undeclared work (i.e., supply side of informal economy) being also reported in Nordic nations [64, 65]. Although surprising at a first sight, this is explained by the higher level of cooperation displayed by the respondents from the Nordic nations compared with the respondents from other European regions.

**Table 2:** Reasons for which the European consumers make purchases from the informal economy: By country (%; N = 27,565).

	Purchasing undeclared goods and services	Reasons:			
		More convenient ^1)^	Lack of availability on regular market	Involuntary (realised afterwards it was informal market)	Social and/or redistributive reasons
*EU-27 + UK*	*10*	*57*	*10*	*16*	*36*
Nordic Nations	14	61	12	20	40
Denmark	16	69	9	8	44
Sweden	13	64	14	21	38
Finland	13	47	13	35	41
Southern Europe	13	61	6	15	37
Malta	30	33	6	52	11
Greece	27	72	11	8	35
Cyprus	16	58	12	22	41
Portugal	16	49	9	13	34
Italy	12	68	5	17	43
Spain	9	45	4	14	31
East-Central Europe	10	65	13	12	33
Latvia	21	64	11	17	24
Croatia	18	74	15	7	45
Bulgaria	17	64	24	13	27
Czech Republic	17	67	10	11	49
Lithuania	16	76	16	20	22
Hungary	15	78	13	8	20
Estonia	13	55	12	21	27
Slovakia	12	64	3	13	50
Slovenia	11	63	11	16	38
Romania	7	48	12	16	35
Poland	5	59	10	9	24
Western Europe	9	50	11	19	36
Netherlands	27	54	17	4	48
Belgium	16	56	11	25	34
Ireland	14	54	18	10	32
Luxembourg	13	55	12	8	45
Austria	12	59	8	7	46
France	8	54	7	18	42
Germany	7	47	11	28	28
United Kingdom	5	40	9	27	24

Notes

^1)^ Lower price and/or faster/better service/product.

*Source*: author`s calculations based on data from Special Eurobarometer 498 –Wave EB92.1, Undeclared Work in the European Union, Fieldwork—September 2019 / Publication date—February 2020 (European Commission, 2021).

Exploring what type of goods and services consumers acquired from the informal economy, the finding is that 30% of participants had purchased home maintenance and improvement services, 27% hairdressing or beauty services, 19% repair services (e.g., mobile phone, car), 17% other goods or services (to those included in the survey), 16% cleaning or ironing, 16% food products (e.g., farm produce), 13% gardening services, 7% babysitting and 7% healthcare services. Similarly, a study investigating 11 East-Central countries, found that 31% of the total purchases in the informal economy are represented by home repairs and renovation purchases [19].

Why do consumers purchase the goods and services they need from the informal economy? Is it more convenient (lower price and/or faster/better service/product)? Or do they do so because they have no other choice considering that the good or service they need is not available on the formal regular market? Or is it the case that consumers are making these purchases for social ends? To answer these questions, those reporting that they have made a purchase form the informal market were asked ‘Why did you buy these goods or services undeclared instead of buying them on the regular market?’ As such, Table 3 reveals that seeking a more convenient deal (lower price and/or faster/better service/product) is the single motive of the consumers in only 46% of cases, one of multiple motives in 23% of informal purchases and not cited at all as a rationale for these type of purchases in the remaining 31% of the cases. Some 5% of purchases in the informal economy are explained exclusively by the lack of availability of the good and/ or service on the formal regular market. In addition, the lack of availability rationale was mentioned as a reason for purchasing goods and services from the informal economy alongside with other motives in 7% of cases. As such, the results show a decline in the motives related to this rational economic actor explanation over time. In 2007, 44% of the consumers in the informal economy make these purchases motivated by financial goals alone and a further 15% due to formal market failures alone [14]. Similarly, when supply-side of the informal economy is analysed, the finding is that there are motives other than the financial necessity (i.e., marginalisation theory) that drive people into the informal realm, with two times more suppliers in the informal economy in the European Union voluntarily choosing to exit the formal market [66] due to different motives such as, for example, burdensome regulation [42–44].

**Table 3:** Combinations of reasons why European consumers make purchases from the informal economy: By region (%; N = 3,018).

	EU-27 + UK	East-Central Europe	Southern Europe	Western Europe	Nordic nations
More convenient^1^ alone	46	51	48	43	42
Lack of availability on regular market	5	5	2	7	7
Social and/or redistributive reasons alone	22	16	21	26	18
Combination of more convenient & social and/or redistributive reasons	20	18	23	17	25
Combination of more convenient & lack of availability on regular market	3	5	3	3	3
Combination of social and/or redistributive reasons & lack of availability on regular market	2	2	1	2	1
Combination of more convenient, social and/or redistributive reasons & lack of availability on regular market	2	3	2	2	4

Notes

^1)^ Lower price and/or faster/better service/product.

*Source*: author`s calculations based on data from Special Eurobarometer 498 –Wave EB92.1, Undeclared Work in the European Union, Fieldwork—September 2019 / Publication date—February 2020 (European Commission, 2021).

Social and/or redistributive motives are the only reason in 22% of all purchases in the informal economy, and in an additional 24% of cases this reason is cumulated with other motives. The finding is therefore, that most of the consumers purchase from informal economy for other reasons than solely convenience in terms or financial gain or better value for the money (better product or service or higher speed of provision). Similarly, a study investigated the supply-side of the informal economy in the European Union member states concluded that the highest proportion of undeclared work was conducted voluntarily, as paid favours, for close social relations [52].

In consequence, in the EU-27 and the UK the consumers’ purchases in the informal economy is not fully explained by one or other of these theoretical explanations. As the survey results show, both theoretical explanations are valid for a certain category of consumer and must be used together to explain consumers’ purchases in the informal economy. Important to mention, nevertheless, is that the importance and the prevalence of each of these theoretical explanations differs across EU regions as Table 3 reveals. For example, the rationale of making this type of purchases for convenience reasons (better price or better value for money due to faster provision or better quality of the purchased good or service) is more prevalent in East-Central Europe (51%) but less prevalent in Western Europe (43%) and the Nordic Nations (42%). Meanwhile, the lack of availability on formal regular market are more often specified as the unique reason in Western Europe and the Nordic nations (7%), while purchases driven purely by social ends rationales are more often mentioned in Western Europe (26%). Similarly, suppliers on the informal economy in Western Europe and Nordic nations engage in this realm mostly voluntarily, for close social relations [52].

Variations on motives for making purchases in the informal economy are noticed as well across consumers groups. These variations are displayed in Table 4 which shows firstly who is the European citizen more likely to make purchases from the informal economy and secondly, what consumer cites which rationale. Starting with the consumer from the informal economy, Model M1 in Table 4 shows that youngest population is more likely than other age groups to purchase from the informal economy and so too are men compared with women. Also, the respondents with a lower tax morality are more likely to participate in informal paid transactions than those displaying a higher tax morality. In terms of occupation, it seems that compared with those unemployed, those in self-employment, managers and with collars are more likely to make such purchases whilst students and house persons are less likely to do so. Similarly, single households are more likely to make such purchases compared with larger households as well as are those respondents having difficulties in paying bills most of the time compared with those who seldom or never encounter such problems. In addition, those with children are more likely than those without children to get involved in informal paid transactions, as well as those living in larger towns.

**Table 4:** Multilevel mixed-effects logistic regression of the probability to purchase from the informal economy in the EU-27 and the United Kingdom.

Fixed part				M1*—Purchasing undeclared goods and services*						
				Coef.		SE		OR		(OR, 95% CI)
Tax morality				-0.175	***	0.009		0.839		(0.824–0.854)
Gender (R: Male)										
Female				-0.131	***	0.039		0.877		(0.813–0.946)
Age (exact age)				-0.009	***	0.002		0.991		(0.988–0.994)
Marital status (R: (Re-)Married/ Living with partner)										
Single				-0.007		0.068		0.993		(0.870–1.134)
Divorced or separated				0.021		0.079		1.021		(0.875–1.192)
Widow/other				-0.123		0.088		0.884		(0.744–1.051)
Occupation (R: Unemployed)										
Self-employed				0.366	***	0.102		1.442		(1.181–1.761)
Managers				0.337	***	0.097		1.400		(1.158–1.692)
Other white collars				0.213	**	0.093		1.238		(1.031–1.486)
Manual workers				-0.068		0.090		0.934		(0.783–1.115)
House persons				-0.254	**	0.121		0.776		(0.612–0.984)
Retired				-0.071		0.100		0.931		(0.765–1.134)
Students				-0.359	***	0.124		0.698		(0.548–0.889)
Difficulties paying bills (R: Most of the time)										
From time to time				-0.207	***	0.071		0.813		(0.708–0.934)
Almost never/ never				-0.239	***	0.070		0.788		(0.687–0.903)
People 15+ years in own household (R: One)										
Two				-0.150	**	0.065		0.861		(0.757–0.978)
Three				-0.189	**	0.076		0.827		(0.713–0.960)
Four and more				-0.215	***	0.083		0.807		(0.686–0.949)
Children (R: No children)										
Having children				0.169	***	0.047		1.185		(1.081–1.298)
Area (R: Rural area or village)										
Small/ middle sized town				0.038		0.047		1.039		(0.949–1.138)
Large town				0.207	***	0.048		1.230		(1.120–1.351)
Region (R: East-Central Europe)										
Western Europe				-0.078		0.239		0.925		(0.579–1.478)
Southern Europe				0.519	**	0.261		1.680		(1.007–2.803)
Nordic Nations				0.129		0.335		1.138		(0.591–2.194)
Constant				0.195		0.219				
Random part										
Country-level variance				0.253***						
(Standard error)				0.071						
Variance at country level (ICC) (%)				7.15						
N				25,631						
Countries				28						
Wald chi2				721.14						
Prob > chi2				0.000						
Fixed part	**M2a** –Reason: *More convenient* ^*1)*^					**M2b –**Reason: *Lack of availability on regular market*				
	Coef.		SE	OR	(OR, 95% CI)	Coef.		SE	OR	(OR, 95% CI)
Tax morality	-0.090	***	0.018	0.914	(0.882–0.948)	0.021		0.026	1.021	(0.970–1.075)
Gender (R: Male)										
Female	-0.228	***	0.074	0.796	(0.689–0.921)	0.032		0.110	1.032	(0.832–1.280)
Age (exact age)	-0.005		0.003	0.995	(0.988–1.001)	0.001		0.005	1.001	(0.991–1.011)
Marital status (R: (Re-)Married/ Living with partner)										
Single	-0.108		0.131	0.898	(0.694–1.161)	-0.227		0.206	0.797	(0.533–1.193)
Divorced or separated	0.260	*	0.154	1.297	(0.959–1.756)	0.328		0.207	1.388	(0.925–2.082)
Widow/other	0.036		0.167	1.036	(0.746–1.439)	0.043		0.251	1.044	(0.638–1.709)
Occupation (R: Unemployed)										
Self-employed	-0.238		0.196	0.788	(0.537–1.157)	0.359		0.270	1.432	(0.844–2.432)
Managers	-0.279		0.186	0.757	(0.526–1.090)	-0.075		0.267	0.928	(0.549–1.566)
Other white collars	-0.141		0.181	0.869	(0.609–1.239)	0.092		0.257	1.096	(0.663–1.813)
Manual workers	-0.089		0.178	0.915	(0.646–1.296)	-0.298		0.259	0.742	(0.446–1.234)
House persons	-0.185		0.234	0.831	(0.525–1.314)	0.070		0.336	1.072	(0.555–2.072)
Retired	-0.225		0.197	0.799	(0.543–1.175)	-0.017		0.283	0.983	(0.564–1.712)
Students	-0.476	**	0.241	0.621	(0.388–0.995)	-0.541		0.413	0.582	(0.259–1.307)
Difficulties paying bills (R: Most of the time)										
From time to time	0.095		0.135	1.100	(0.844–1.435)	0.215		0.206	1.239	(0.828–1.855)
Almost never/ never	-0.167		0.132	0.846	(0.653–1.097)	0.094		0.204	1.099	(0.737–1.638)
People 15+ years in own household (R: One)										
Two	-0.109		0.126	0.897	(0.701–1.148)	0.283		0.189	1.327	(0.916–1.923)
Three	-0.081		0.145	0.922	(0.694–1.224)	0.348		0.218	1.416	(0.923–2.172)
Four and more	-0.149		0.156	0.861	(0.634–1.170)	0.240		0.240	1.271	(0.793–2.036)
Children (R: No children)										
Having children	-0.089		0.089	0.915	(0.768–1.089)	0.131		0.129	1.140	(0.885–1.469)
Area (R: Rural area or village)										
Small/ mid-sized town	0.016		0.089	1.016	(0.854–1.210)	0.039		0.131	1.039	(0.804–1.344)
Large town	-0.120		0.091	0.887	(0.742–1.060)	0.072		0.133	1.074	(0.828–1.393)
Region (R: East-Central Europe)										
Western Europe	-0.470	**	0.187	0.625	(0.433–0.901)	-0.107		0.165	0.898	(0.650–1.242)
Southern Europe	-0.367	*	0.203	0.693	(0.466–1.031)	-0.570	***	0.191	0.566	(0.389–0.822)
Nordic Nations	-0.173		0.259	0.841	(0.507–1.396)	-0.181		0.235	0.834	(0.526–1.323)
Constant	2.144	***	0.322			-2.504	***	0.446		
Random part										
Country-level variance					0.118					0.041
(Standard error)					0.042					0.030
Variance at country level (ICC) (%)					3.47					1.24
N					3,637					3,637
Countries					28					28
Wald chi2					81.79					42.34
Prob > chi2					0.000					0.012
Fixed part	**M2c** –Reason: *Involuntary (realised afterwards it was informal market)*					**M3 –**Reason: *Social and/or redistributive reasons*				
	Coef.		SE	OR	(OR, 95% CI)	Coef.		SE	OR	(OR, 95% CI)
Tax morality	0.220	***	0.030	1.247	(1.176–1.321)	-0.055	***	0.018	0.946	(0.914–0.980)
Gender (R: Male)										
Female	-0.001		0.102	0.999	(0.817–1.220)	-0.085		0.075	0.919	(0.793–1.065)
Age (exact age)	-0.002		0.005	0.998	(0.989–1.007)	-0.002		0.003	0.998	(0.991–1.005)
Marital status (R: (Re-)Married/ Living with partner)										
Single	0.104		0.179	1.109	(0.782–1.574)	-0.178		0.133	0.837	(0.645–1.087)
Divorced or separated	0.091		0.211	1.095	(0.725–1.655)	0.061		0.151	1.063	(0.791–1.428)
Widow/other	0.212		0.228	1.236	(0.790–1.934)	0.199		0.169	1.221	(0.876–1.702)
Occupation (R: Unemployed)										
Self-employed	-0.390		0.267	0.677	(0.401–1.141)	-0.030		0.189	0.970	(0.670–1.405)
Managers	-0.209		0.247	0.811	(0.500–1.317)	-0.180		0.182	0.835	(0.585–1.193)
Other white collars	-0.229		0.241	0.796	(0.496–1.276)	-0.129		0.175	0.879	(0.624–1.238)
Manual workers	-0.416	*	0.239	0.660	(0.413–1.053)	-0.015		0.170	0.985	(0.706–1.375)
House persons	0.051		0.305	1.052	(0.578–1.915)	0.000		0.234	1.000	(0.632–1.581)
Retired	-0.447	*	0.267	0.639	(0.379–1.079)	-0.182		0.192	0.833	(0.572–1.215)
Students	-0.349		0.320	0.706	(0.377–1.322)	-0.144		0.241	0.866	(0.540–1.389)
Difficulties paying bills (R: Most of the time)										
From time to time	0.363	*	0.204	1.437	(0.963–2.145)	-0.295	**	0.133	0.744	(0.574–0.966)
Almost never/ never	0.438	**	0.200	1.549	(1.047–2.292)	-0.311	**	0.131	0.733	(0.567–0.948)
People 15+ years in own household (R: One)										
Two	0.287	*	0.171	1.333	(0.953–1.865)	-0.015		0.126	0.985	(0.769–1.261)
Three	0.079		0.201	1.082	(0.730–1.606)	-0.064		0.146	0.938	(0.705–1.249)
Four and more	0.275		0.212	1.316	(0.869–1.994)	-0.082		0.158	0.921	(0.675–1.256)
Children (R: No children)										
Having children	-0.112		0.123	0.894	(0.703–1.137)	0.086		0.090	1.090	(0.914–1.300)
Area (R: Rural area or village)										
Small/ mid-sized town	0.153		0.124	1.166	(0.914–1.486)	-0.071		0.089	0.932	(0.782–1.110)
Large town	0.111		0.127	1.117	(0.872–1.432)	-0.151		0.093	0.860	(0.717–1.031)
Region (R: East-Central Europe)										
Western Europe	-0.039		0.310	0.962	(0.523–1.767)	0.381	*	0.205	1.463	(0.980–2.186)
Southern Europe	0.238		0.332	1.268	(0.661–2.433)	0.046		0.224	1.047	(0.675–1.623)
Nordic Nations	0.391		0.423	1.478	(0.645–3.384)	0.530	*	0.282	1.699	(0.977–2.955)
Constant	-3.907	***	0.473			0.203		0.318		
Random part										
Country-level variance					0.352					0.148
(Standard error)					0.114					0.052
Variance at country level (ICC) (%)					9.67					4.32
N					3,637					3,637
Countries					28					28
Wald chi2					78.58					41.67
Prob > chi2					0.000					0.014

Notes

*** p<0.01

** p<0.05

* p<0.1; Coef. = Coefficient; SE = Standard Error; OR = Odds Ratio; CI = Confidence Interval. The coefficients displayed are compared with the reference category displayed in brackets (R). Only individuals which responded and have data for each and every control variable were kept in the analysis.

^1)^ Lower price and/or faster/better service/product.

*Source*: author`s calculations based on data from Special Eurobarometer 498 –Wave EB92.1, Undeclared Work in the European Union, Fieldwork—September 2019 / Publication date—February 2020 (European Commission, 2021).

Variegated results in relation with the influence of individual`s socio-demographic characteristics were also reported in relation to the supply-side of the informal economy. However, similar to the demand side of the informal economy, the results on the supply side reveal the pivotal role of tax morale. As such, tax morale has been identified as being associated with the prevalence of various non-compliant behaviours such as: participating in the shadow economy [67, 68], tax evasion [69], engaging in the supply-side of the informal economy [70], under-reporting salaries [5, 6] or making informal payments to practitioners in the public healthcare sector [59–62].

Models M2 and M3 in Table 4 display that there are differences in the type of people who are more likely to mention each driver for acquiring informal good and services. As Model M2a display, consumers who are more likely to cite motives related with convenience, and thus those consumers weighting the cost/benefit ratio, are men, those with a lower tax morality and those living in East-Central Europe.

Examining in Model M2b in Table 4 who is driven in the informal economy by the lack of the availability of the desired good or service on the regular formal market it seems that there is no statistically significant variation across consumers groups. The only significant variation displayed is between regions, with those living in Southern Europe being significantly less likely than those living in East-Central Europe to make purchases from the informal market due to the fact they have no other choice considering that the service or the good they need is not available on the regular formal market. This result is not surprising and shows that the personal characteristics are not important when the consumer is left no other choice.

Turning to those who involuntarily made purchases from the informal economy, and as Model M2c in Table 4 displays, it seems that those with a high tax morality are more likely to cite this reason (doubtless because of their own high tax morale they did not question the morale of the seller) as well as those who are financially better off and never or almost never encounter difficulties in paying their household bills.

**Fig 1:**
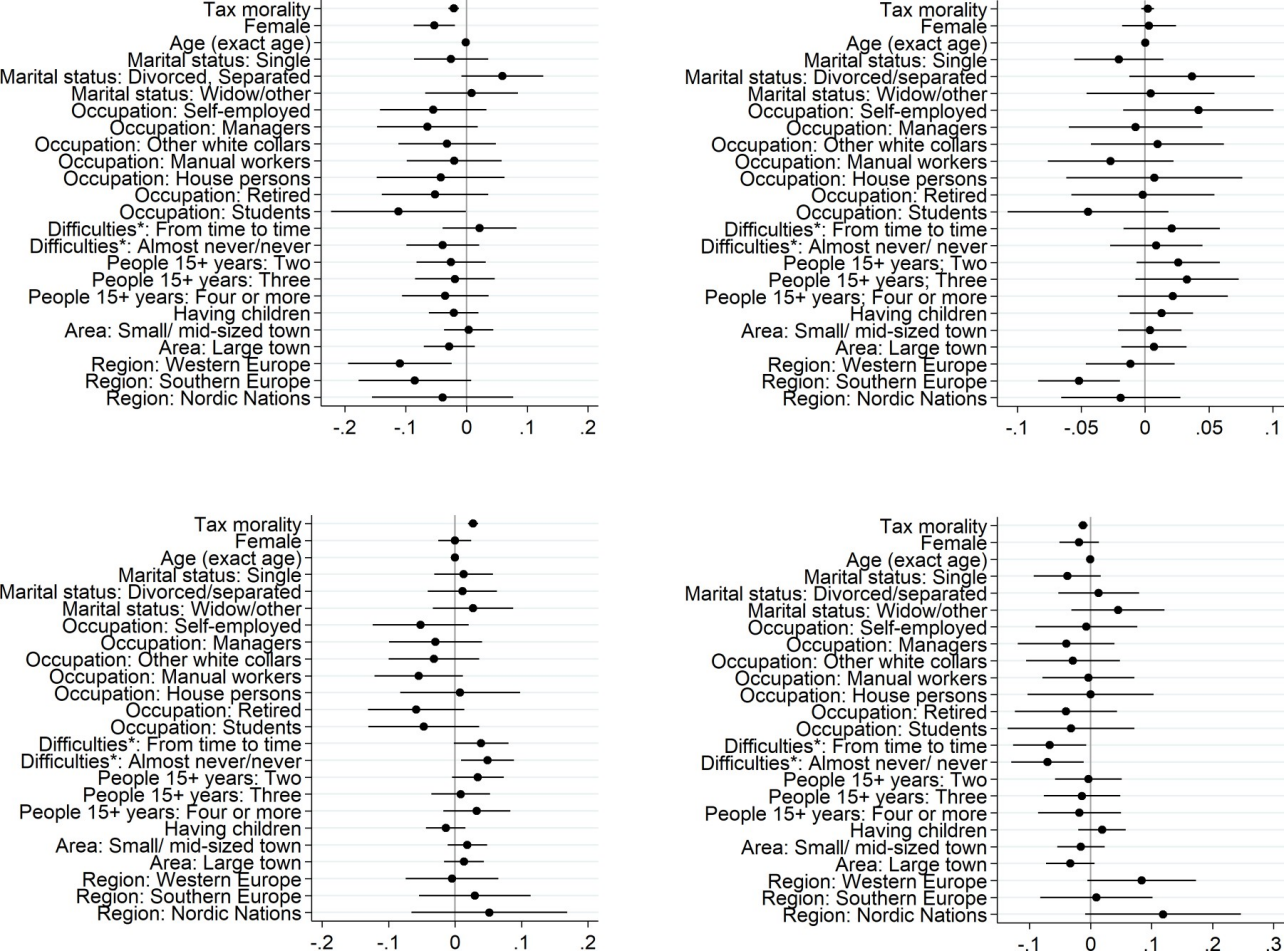
Marginal effects. A. More convenient^1^) , B. Lack of availability on regular market, C. Involuntary, D. Social and/or redistributive reasons. *Notes*: ^1)^ Lower price and/or faster/better service/product; * Difficulties in paying (household) bills. *Source*: Author`s calculations based on data from Special Eurobarometer 498 –Wave EB92.1, Undeclared Work in the European Union, Fieldwork—September 2019 / Publication date—February 2020 (European Commission, 2021).

Finally, evaluating the social actor theoretical explanation that people make purchases in the informal economy for social ends, Model M3 in Table 4 reveals that those purchasing informally to help someone are those with low tax morality, experiencing themselves difficulties in paying the household bills, living rather in Western Europe or Nordic nations. To enable better understanding of the magnitude of these associations between the socio-characteristics of the consumer and the likelihood of purchasing from informal economy for each analysed motive, the marginal effects for Models M2 and M3 are displayed in Fig 1.

**Fig 2:**
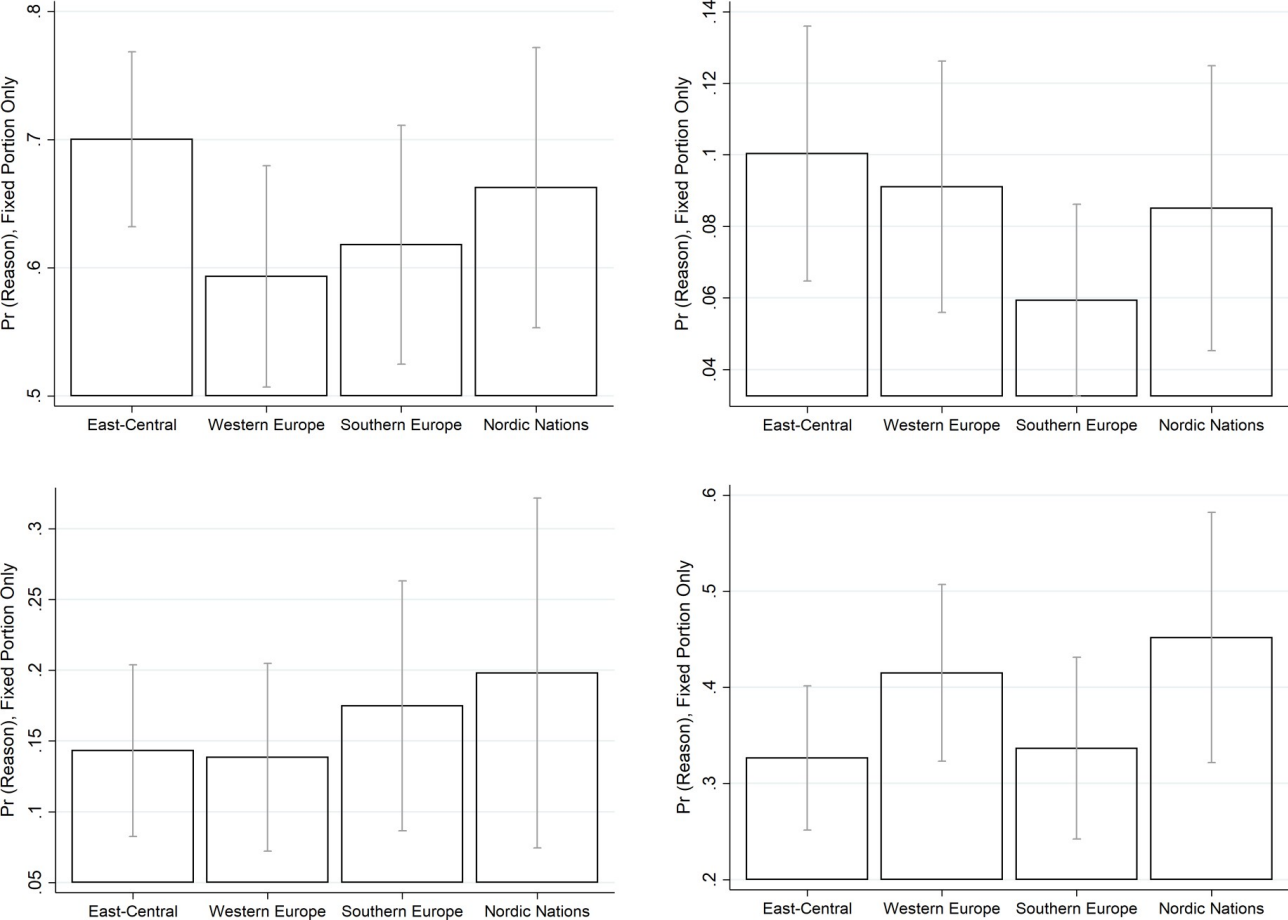
Predicted probabilities of reasons for a ‘representative consumer’ to make purchases from the informal economy: By region (with 95% CI). A. More convenient ^1^) B. Lack of availability on regular market, C. Involuntary, D. Social and/or redistributive reasons. *Notes*: ^1)^ Lower price and/or faster/better service/product. *Source*: author`s calculations based on data from Special Eurobarometer 498 –Wave EB92.1, Undeclared Work in the European Union, Fieldwork—September 2019 / Publication date—February 2020 (European Commission, 2021).

**Fig 3:**
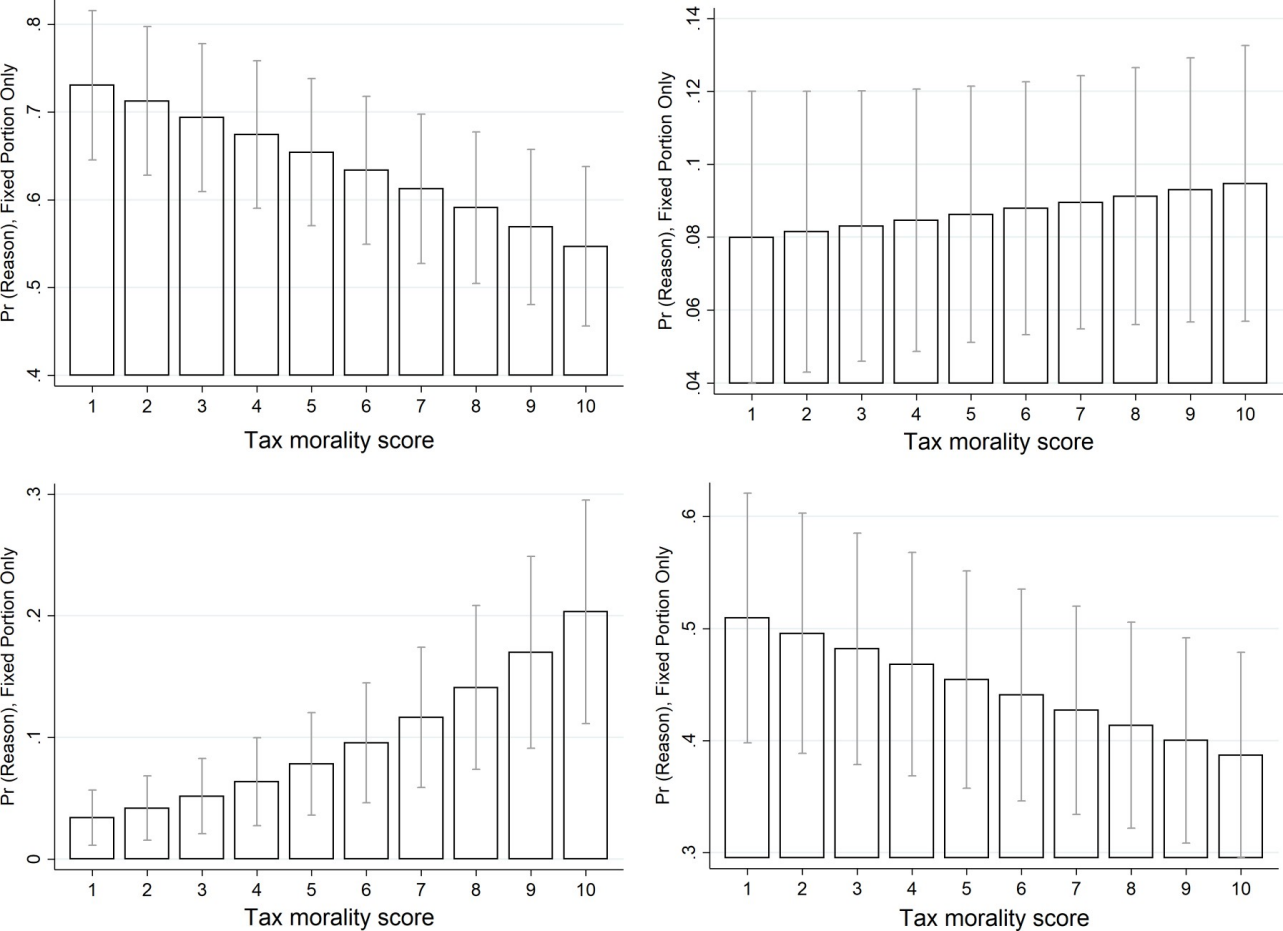
Predicted probabilities of reasons for a ‘representative consumer’ to make purchases from the informal economy: By level of tax morality (with 95% CI). A. More convenient ^1^) B. Lack of availability on regular market, C. Involuntary, D. Social and/or redistributive reasons. *Notes*: Higher tax morality score = high tax morality; ^1)^ Lower price and/or faster/better service/product. *Source*: author`s calculations based on data from Special Eurobarometer 498 –Wave EB92.1, Undeclared Work in the European Union, Fieldwork—September 2019 / Publication date—February 2020 (European Commission, 2021).

To enable understanding of the variations in drivers of purchases from the informal economy, Figs 2 and 3 graphically portray the ‘representative’ consumer in the informal economy by EU region and tax morality levels. As Fig 2 reveals, if living in East-Central Europe the representative consumer is more likely to be driven to the informal economy due to convenience reasons (better price and/or faster and better quality good or service) or lack of availability of the needed product or service on the regular formal market. Meanwhile, the representative consumer living in Nordic nations or Southern Europe is more likely to have made purchases in the informal economy involuntarily, realising only after the purchase has been made that it belonged to the informal economy. Finally, this representative consumer is more likely to mention social and/or redistributive motives for their engagement in informal economy if living in Nordic nations or Western Europe.

Important to stress is also the fact that as Table 4 displayed, tax morality appears to be a significant determinant of whether consumers make purchases in the informal economy. In recent years, this has begun to be widely recognised, albeit until now mostly about those working in the informal economy [5, 71, 72]. Grounded in institutional theory [73], it is argued that where formal institutions or the ‘state morality’ and informal institutions or ‘civic morality’ are not aligned—asymmetry which is measured by the level of tax morality—individuals are found to be more prone to informal economy as a supplier (i.e., undertake undeclared work). Until now, this has been under-investigated from the demand-side perspective. Table 4, however, reveals that this is similarly the case for the demand-side; the lower the tax morale of consumers, the more likely they are to acquire goods and services from the informal economy, and this is a statistically significant correlation. Here, therefore, we examine this relationship in more depth by analysing the how the predicted probability of consumers to purchases from the informal economy driven by each analysed reason varies by their tax morale score. As Fig 3 shows, a representative EU consumer displaying a lower tax morale is far more likely to cite the convenience rationale (lower price or better value for money in terms of speed and quality of the purchased goods and services) or social and redistributive rationales as their drivers of purchasing informally compared with the representative consumer with higher tax morale. As for lack of availability on the regular market, the results are less clear cut, with small differences. Interesting, however, is that when examining the involuntary purchases, it is those with higher tax morale who are more likely to cite this as the reason for purchasing informally. Indeed, this seems appropriate. Those with higher tax morale seem likely to make purchases from the informal economy only if the goods and services are not available on the regular market or if they do not know that the purchase they made belongs to the informal economy, whilst those with lower tax morality motivated more of the reason of convenience or social and redistributive reasons.

## 5. Discussion

In this paper, we have sought to advance the knowledge on the consumer behaviour on the informal economy by reporting contemporary data, namely the results of a 2019 Eurobarometer survey [55] which involved 27,565 respondents from 27 European Union member states and the UK. The results of the multilevel mixed effects logit regression analysis has revealed that men, younger age groups, those living in one person households, in large towns, those with children, having financial difficulties as well as those displaying a low tax morality are more likely than other groups to make purchases from the informal economy. To evaluate their reasons for doing so, two major potential explanations have been evaluated. Firstly, there is the view of consumers as rational economic actors choosing the better possible deal (lower price and/or better value for money in terms of faster provision or better quality of the good or service), acquiring from the informal market because the good or service they need is not available on the regular market or making this purchase involuntarily, realising that they made a purchase from the informal sector only afterwards. Secondly, consumers are seen as social actors making such purchases for helping a person from their close networks.

The descriptive statistics reveal that convenience (i.e., lower price and/or faster/better service/product) is the unique motive in only 46% of the purchases, one of several motives in an additional of 23% of the purchases and not mentioned at all in a further 31% of cases. As such, in 54% of the transactions, other motives are involved, beyond seeking a more convenient deal. Consumers make this type of undeclared paid transaction therefore, for other reasons such as being provided no option on the regular market, participating in the informal economy involuntarily or doing so to help a person from a close network (i.e., social reasons). Indeed, about 16% of those involved in undeclared paid transactions did so unintentionally by not being aware that the purchase they made belongs to the informal economy. The prevalence of each of these theoretical explanations, as this study revealed, varies across population groups and EU regions.

The theoretical implications of the findings are twofold. Firstly, they reveal the need to transcend the profit maximisation view according to which the consumer behaviour is a result of the balance between the costs and the benefits of a certain activity such as purchasing from the informal sector in this case. The results reveal that consumers often participate to paid undeclared transactions to help someone from their close networks (i.e., social motives), because they cannot find the good or service they need on the regular market or simply because they are not aware that they make purchases from the informal market. Secondly, this study reveals how these competing explanations are not mutually exclusive. Indeed, quite the opposite is the case. In many instances, the consumer was driven to the informal economy due to a mixture of motives and not a sole motive. Therefore, all these theoretical perspectives need to be used to be able to fully explain consumer purchases in the informal economy, albeit with their relative importance varying according to socio-demographic and spatial characteristics.

## 6. Conclusions

The findings of this study also have important policy implications. On the one hand, they reveal the group of consumers who should be targeted first when tackling undeclared paid transactions, namely: men, younger age groups, those living in single households, in large towns, those having children and those with a lower tax morality. Indeed, educational and awareness campaigns started to be implemented at the national or even Europe level, albeit so far, they are mainly targeted at the supply side of the informal economy, on those undertaking undeclared work (see for example #EU4FairWork campaign or Rights for all Season campaign of the European Commission`s European Platform tackling undeclared work). For the consumer of goods and services from the informal market, these campaigns could focus on improving their tax morale, which was shown to be closely correlated with these informal transactions and focus on benefits of purchasing goods and services from the formal sector and the costs of acquiring them from the informal economy (e.g., lack of insurance, lack of guarantee that the required health and safety standards are met).

On the other hand, the results are also useful in deciding what policy measures are required to tackle such consumption. The results indicate that the consumers are not participating into informal economy solely due to profit maximisation motives (i.e., lower price or better value for money), and therefore the deterrence policy measures aimed at altering the cost-benefit ratio of engaging in such transitions by increasing the penalties needs to be accompanied by policy measures that aim to increase the benefits of operating in the formal economy or to even provide incentives for attracting the consumer to the formal economy. These include amnesties, voluntary disclosure measures and tax incentives to encourage consumers to purchase formal goods and services or to request the receipts, such as service vouchers or fiscal receipts lotteries [1, 74]. Such policies applied in practice, which could serve as good practices for other nations include: tax rebates for purchasers in Sweden and Denmark, lotteries of fiscal receipts in Croatia and Romania, service vouchers in Austria, Belgium and France, and holiday vouchers in Romania [75, 76].

However, the results of the study display that the policy measures aimed at altering the cost-benefit ratio either by increasing the penalties for operating in the informal sector or by increasing the attractiveness of the formal sector will not be always effective because consumers have various other motives for engaging in the informal economy, beyond the result of weighing up the costs and benefits of doing so. Purchases in the informal economy are also a result of consumers trying to help a person in need from their close networks as well because of lack of other choices because they cannot find the good or the service they need on the regular market or even because they participate in this type of transactions on an involuntary basis.

To tackle consumption in the informal economy, in consequence, the provision of certain goods and services needs to be improved as well as some measures aimed at those making such purchases for social motives need to be implemented. The issue of formal goods and services provision can be addressed by organising one-stop shops or regular trade fairs, developing sharing economy platforms, creating apps and local hot-lines to enable contact between the customers and formal sector suppliers. Such instruments help the suppliers to advertise their goods and services, provide a formal solution for the customers and protect them ensuring that the goods and services meet the health and safety regulations in place. On the second issue of tackling transactions aimed at social ends, policy measures can include education and awareness raising campaigns to highlight the benefits of operating in the formal economy for all concerned. They might also include policy initiatives to legitimise the small jobs undertaken for social ends that are currently undertaken in the informal economy. This could be achieved by implementing a threshold amount that could be earned from small jobs tax free. Such amounts currently exist in some countries. In the UK where there is a 1000 GBP tax free allowance, in Belgium there is a 2,500 EUR tax-free allowance, in France activities under 3,000 EUR or 20 transactions per year are not taxed even if it is necessary to declare these transactions to the state authorities and in Austria the self-employees do not have to declare income less than 720 EUR per month for ‘on the side’ platform activities [77, 78]. Another policy solution is to implement new institutions to allow community help paid exchanges to take place legitimately, such as time banks or Local Exchange and Trading Schemes [79, 80].

In sum, despite the extensiveness of the informal economy globally, little research has been conducted on the behaviour and motives of consumers making purchases in this realm. This paper has begun to fill that gap by providing a contemporary analysis of the consumer of goods and services in the informal economy and their drivers. This reveals the need to adopt a nuanced theorisation which considers the multifarious motives of this behaviour across different consumer groups. If this now leads to an evaluation of the wider applicability of this more variegated theorisation, then one of the major intentions of this paper will have been achieved. If this is then followed by a fuller evaluation of the diversity of policy measures needed to tackle consumers making purchases in the informal economy, then the paper will have achieved its wider purpose. What is for certain, however, is that consumer research theory can no longer solely focus upon consumer behaviour in the formal economy and ignore consumers making purchases in the informal economy.

## Supporting information

S1 Appendix (DOC)
