# Supplementary material for: Explaining consumer motives to purchase in the informal economy
Source: PLoS One. 2021 Oct 15;16(10):e0258686. doi: 10.1371/journal.pone.0258686 (PMC8519443; doi:10.1371/journal.pone.0258686)
Supplement: S1 Appendix — (DOC) [file pone.0258686.s001.doc]

**S1 Appendix**

**S1 Table.** Variables used in the analysis: definitions and descriptive statistics

| Variables | Type | Definition | Mode or mean | | Min/ Max |
| --- | --- | --- | --- | --- | --- |
|  |  |  | N = 25,631 | N = 3,637 |  |
| *Dependent variables* |  |  |  |  |  |
| Purchase of informal goods and services | Dummy | Whether or not the respondent purchased from the informal market in the past 12 months | Not purchased informal goods and services (89%) | - | 0 / 1 |
| More convenient | Dummy | Whether or not the respondent purchased from informal market due to convenience (lower price and/or faster/better service/product) | - | *Lower price* mentioned (57%) | 0 / 1 |
| Lack of availability on regular market | Dummy | Whether or not the respondent purchased from informal market due to lack of availability on formal regular market | - | *Lack of availability on regular market* not mentioned (90%) | 0 / 1 |
| Involuntary (realised afterwards it was informal market) | Dummy | Whether or not the respondent purchased from informal market in an involuntary basis (realised afterwards it was informal market) | - | *Involuntary* not mentioned (84%) | 0 / 1 |
| Social and/ or redistributive reasons | Dummy | Whether or not the respondent purchased from informal market due to social and/ or redistributive reasons | - | *Social and/ or redistributive reasons* not mentioned (64%) | 0 / 1 |
| *Independent variables* |  |  |  |  |  |
| Tax morality | Interval | Average score of self-reported acceptance of undeclared activities | 8.6 | 7.9 | 1 / 10 |
| Gender | Dummy | Respondent`s gender | Female (51%) | Male (53%) | 0 / 1 |
| Age | Interval | Respondent`s age | 49 years | 45 years | 15 / 98  15 / 91 |
| Marital status | Categorical | Respondent`s marital status | (Re-)Married/ Living with partner (64%) | (Re-)Married/ Living with partner (64%) | 1 / 4 |
| Occupation | Categorical | Respondent1s occupation | Retired (27%) | Manual workers (19%) | 1 / 8 |
| Difficulties paying bills | Categorical | Encountering financial difficulties | Almost never/ never (70%) | Almost never/ never (63%) | 1 / 3 |
| People 15+ years in own household | Interval | People aged 15 years or more in respondent`s household | Two (49%) | Two (48%) | 1 / 4 |
| Children | Categorical | Whether or not there is a children (up to 14 years old) in respondent`s household | No children (62%) | No children (57%) | 0 / 1 |
| Area | Categorical | Type of area where the respondent lives | Small or middle sized town (43%) | Small or middle sized town (43%) | 1 / 3 |
| Region | Categorical | EU Region where the respondent lives | Western Europe (51%) | Western Europe (43%) | 1 / 4 |

*Notes:* Only individuals which responded and have data for each and every control variable were kept in the analysis.

*Source:* author`s calculations based on data from Special Eurobarometer 498 – Wave EB92.1, Undeclared Work in the European Union, Fieldwork - September 2019 / Publication date - February 2020 (European Commission, 2021)
